# Vasoactive Intestinal Peptide (VIP) Protects Nile Tilapia (*Oreochromis niloticus*) against *Streptococcus agalatiae* Infection

**DOI:** 10.3390/ijms232314895

**Published:** 2022-11-28

**Authors:** Zhiqiang Zhang, Qi Li, Yongxiong Huang, Zhou Xu, Xinjin Chen, Baijian Jiang, Yu Huang, Jichang Jian

**Affiliations:** 1 Guangdong Provincial Key Laboratory of Aquatic Animal Disease Control and Healthy Culture, College of Fishery, Guangdong Ocean University, Zhanjiang 524088, China; 2 Laboratory for Marine Biology and Biotechnology, Qingdao National Laboratory for Marine Science and Technology, Qingdao 266071, China; 3 Guangdong Provincial Engineering Research Center for Aquatic Animal Health Assessment, Shenzhen 327005, China

**Keywords:** Nile tilapia, *Streptococcus agalatiae*, vasoactive intestinal peptide (VIP), vasoactive intestinal polypeptide receptor 1 (VIPR1), immune response, fish

## Abstract

Vasoactive intestinal peptide (VIP), a member of secretin/glucagon family, is involved in a variety of biological activities such as gut motility, immune responses, and carcinogenesis. In this study, the VIP precursor gene (*On-VIP*) and its receptor gene VIPR1 (*On-VIPR1*) were identified from Nile tilapia (*Oreochromis niloticus*), and the functions of On-VIP in the immunomodulation of Nile tilapia against bacterial infection were investigated and characterized. *On-VIP* and *On-VIPR1* contain a 450 bp and a 1326 bp open reading frame encoding deduced protein of 149 and 441 amino acids, respectively. Simultaneously, the transcript of both *On-VIP* and *On-VIPR1* were highly expressed in the intestine and sharply induced by *Streptococcus agalatiae*. Moreover, the positive signals of On-VIP and On-VIPR1 were detected in the longitudinal muscle layer and mucosal epithelium of intestine, respectively. Furthermore, both in vitro and in vivo experiments indicated several immune functions of On-VIP, including reduction of *P65*, *P38*, *MyD88*, *STAT3*, and *AP1*, upregulation of *CREB* and *CBP*, and suppression of inflammation. Additionally, in vivo experiments proved that On-VIP could protect Nile tilapia from bacterial infection and promote apoptosis and pyroptosis. These data lay a theoretical basis for further understanding of the mechanism of VIP guarding bony fish against bacterial infection.

## 1. Introduction

Vasoactive intestinal peptide (VIP), a short peptide containing 28 amino acids belonging to the secretin-glucagon family, is initially isolated from the gastrointestinal tract as a potent vasodilator peptide [1]. The structure of VIP was similar to trypsin, pituitary adenylate cyclase activated polypeptide (PACAP), and glucagon, and particularly the homology between PACAP and VIP was more than 68% [2]. VIP was initially identified in normal nervous tissue and neurons and was subsequently recognized as a neurotransmitter widely distributed in various tissues [3,4]. The wide distribution of VIP determines its involvement in a range of biological activities, such as gut motility, hormonal regulation, circadian rhythms, immune responses, and carcinogenesis [5,6].

In addition, VIP was expressed in various immune cells and participated in the immune response through three G-protein-coupled receptors (GPCRs), namely, VPAC1, VPAC2, and PAC1 [7,8]. VIP receptors (VPAC1 and VPAC2) could bind both VIP and PACAP, then activated the cAMP-PKA pathway, while the PAC1 receptor also activated PKA and PLC [7,9]. The function of VIP receptors in vertebrates (e.g., frog, fish, mouse, chicken, rat, and human) has been well-studied, such as regulating the normal immune functions and affecting the activation of signaling pathways [10]. VPAC1 and VPAC2 are detected in macrophages, CD4^+^ T cells, and CD8^+^ T cells [11,12,13]. In contrast to the VPAC2, which was only expressed after stimulation, the VPAC1 was abundantly expressed in both stimulated and unstimulated lymphocytes and macrophages [10,14].

Commonly, VIP can rapidly react to the most toxic and intense stimulus, which also plays a pivotal role in physiology and pathology. To date, many studies have identified VIP and GPCRs in mammals and indicated that it plays a crucial part in neuro regulation, response to foreign stimuli, and maintenance of autoimmune balance [6,15]. Moreover, VIP and receptors were also identified in some fish and their roles in reproduction were recorded [16,17,18]. However, knowledge about the precise immunomodulation roles and mechanisms of VIP and receptors in bony fish is still lacking.

Nile tilapia (*Oreochromis niloticus*), a fish that is commercially significant in over 100 nations and regions, is a species that is very important for the world’s aquaculture and widely farmed for international trade [19,20]. However, the outbreak of *Streptococcus agalatiae* has resulted in vast losses in the tilapia industry over the past decade and severely influenced the development of tilapia farming in China and around the world [20,21]. Therefore, this study aims to uncover the functions of VIP on the responses of Nile tilapia against *S. agalatiae*. In this study, VIP and VIPR1 gene from Nile tilapia were identified and characterized. Moreover, the expression profiles and immunological roles of VIP under *S. agalatiae* infection were assessed. These data will be the first to shed light on the roles of the secretin-glucagon family in fish innate immunity.

## 2. Results

### 2.1. Sequence Analyses of On-VIP and On-VIPR1

**Figure 1:**
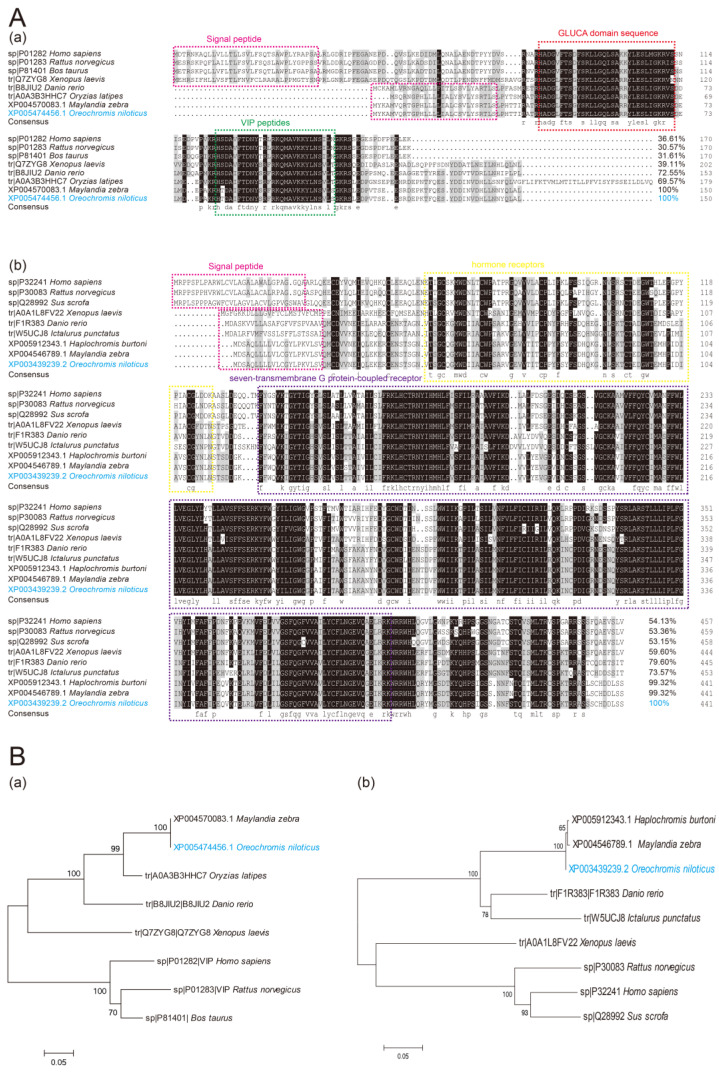
(**A**) The multiple alignments sequence of VIP (**a**) and VIPR1 (**b**) from several species. The signal peptide domain was marked in the pink dotted box. The GLUCA domain sequence and VIP peptides are marked in red and green dotted boxes, and the hormone receptors and seven-transmembrane G protein-coupled receptors are marked in yellow and purple dotted boxes. (**B**) The phylogenetic trees of VIP and VIPR1 were created using the MEGA 6.0 software. On-VIP (**a**) and On-VIPR1 (**b**) are marked in blue.

On-VIP includes a 450 bp ORF, which was thought to encode a potential protein with 149 amino acids and a signal peptide domain (1–29 amino acids). The On-VIP predicted molecular weight was 17.18 kDa and its theoretical p*I* was 8.99. The results of multiple sequence alignments for VIP implied that the sequence of On-VIP contained many highly conserved sequences, including GLUCA domain sequence and vasoactive intestine peptide domain (VIP). Additionally, the BLAST analysis revealed that the sequence of On-VIP was homologous with other VIP. In particular, On-VIP shared more than 69% of its genetic structure with other fish and over 31% with mammals (Figure 1A(a)). Phylogenetic analysis showed that On-VIP distinctly clustered with *Maylandia zebra* and *Oryzias latipes* (Figure 1B(a)).

On-VIPR1 has a 1326 bp ORF that purportedly codes for a potential protein with 441 amino acids and a signal peptide and seven transmembrane domains. The predicted p*I* of On-VIPR1 was 7.78, and its probable molecular weight was 50.18 kDa. Seven extremely standpat transmembrane domains were shown in the results of multiple sequence alignments for VIPR1. In addition, BLAST results showed that the On-VIPR1 sequence was homologous to other VIPR1. Specifically, On-VIPR1 was more than 73% of its genetic structure to other fish and over 53% to mammals (Figure 1A(b)). Phylogenetic analysis showed that On-VIPR1 distinctly clustered with *Maylandia zebra* and *Haplochromis burtoni* (Figure 1B(b)).

### 2.2. Expression Pattern Analyses of On-VIP and On-VIPR1 Amongst Different Tissues

**Figure 2:**
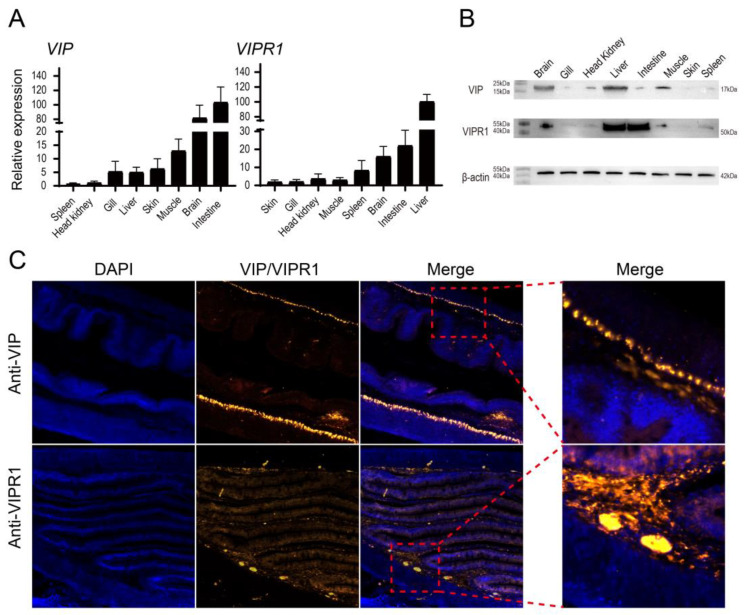
(**A**) By using qRT-PCR, the transcription level of On-VIP and On-VIPR1 in various organs of unchallenged and healthy tilapia was identified. Every value is presented as a mean and standard deviation; n = 3. The transcriptional levels of On-VIP and On-VIPR1 in the spleen and skin were set as 1, respectively. (**B**) By using Western blot, the protein level of On-VIP and On-VIPR1 in various organs of unchallenged and healthy tilapia were assessed. (**C**) The location of On-VIP and On-VIPR1 in the intestine of unchallenged and healthy tilapia. The results were observed using the ZEISS Axioscope 5 microscope with × 200 and × 400 magnifications (Zeiss, Jena, Germany).

The relative expression patterns of On-VIP and On-VIPR1 in various organs of unchallenged and healthy tilapia were examined using qRT-PCR. The relative expression of *On-VIP* was high in the intestine and brain, followed by muscle, skin, and liver, and lowest in spleen. The expression of *On-VIPR1* was high in liver and intestine, followed by brain, spleen, and muscle, and lowest in skin (Figure 2A).

Western blot was applied to assess the expression patterns of On-VIP and On-VIPR1 at protein levels in unchallenged and healthy tilapia. The protein level of On-VIP was high in liver and brain, followed by muscle and head kidney, and the lowest was observed in spleen. The protein level of On-VIPR1 was high in liver and intestine, followed by brain and muscle, and the lowest in skin (Figure 2B).

Additionally, fluorescent immunohistochemistry was applied to detect the distribution patterns of On-VIP and On-VIPR1 in healthy tilapia intestine. The remarkable positive signals of On-VIP were observed in the longitudinal muscle layer of healthy tilapia intestine, while On-VIPR1 was predominantly detected in the mucosal epithelium of healthy tilapia intestine (Figure 2C).

### 2.3. Expression Pattern Analyses of On-VIP and On-VIPR1 during Bacterial Challenge

**Figure 3:**
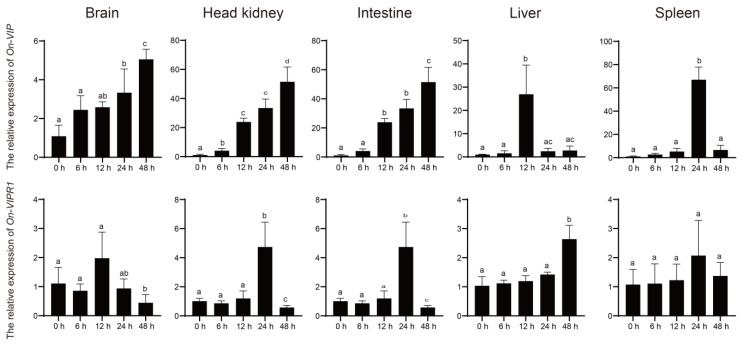
The transcriptional levels of *On-VIP* and *On-VIPR1* in the liver, head kidney, brain, spleen, and intestine of tilapia infected with *S. agalactiae* at various time points via qRT-PCR. The transcriptional levels of *On-VIP* and *On-VIPR1* at 0 h were set as 1. Every value was presented as the mean and standard deviation; n = 3. The different letters were applied to reveal the significant difference (*p* < 0.05).

After infection with *S. agalactiae*, the expression of *On-VIP* increased following a time-dependent pattern in brain, head kidney, and intestine. Moreover, the expression levels of *On-VIP* peaked in liver and spleen at 12 h and 24 h, respectively. Meanwhile, the transcription of *On-VIPR1* peaked at 24 h and 48 h in head kidney, intestine, and liver. However, the transcription of *On-VIPR1* was not altered by *S. agalactiae* in spleen (Figure 3).

### 2.4. Effects of On-VIP on Inflammatory Factors in Tilapia MO/MΦ

**Figure 4:**
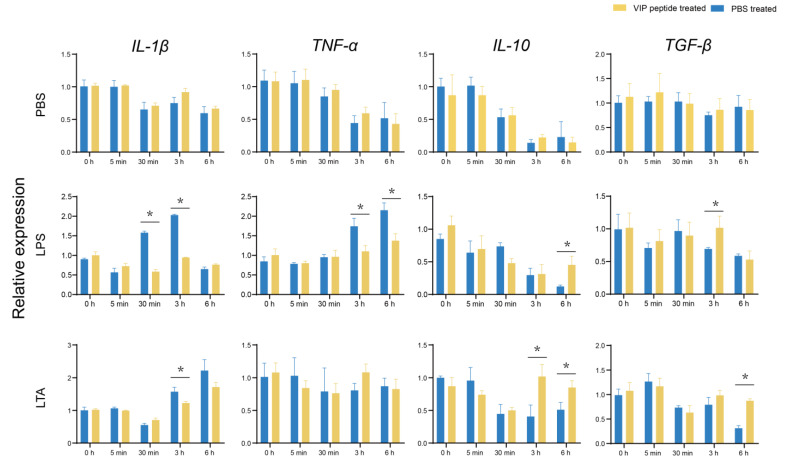
The relative expression of inflammatory-related factors (*IL-1β*, *TNF-α*, *IL-10*, and *TGF-β*) after LPS and LTA challenged of tilapia MO/MΦ was analyzed using qRT-PCR. Every value is presented as the mean and standard deviation; n = 3. The asterisks reveal the significant difference (*p* < 0.05).

The expression of *IL-1β* and *TNF-α* in the LPS + VIP + MO/MΦ group markedly decreased (*p* < 0.05) after LPS challenged for 3 h and 6 h, respectively, compared with the those in the LPS + PBS + MO/MΦ group. In addition, the transcriptional levels of *IL-10* and *TGF-β* in the LPS + VIP + MO/MΦ group increased substantially (*p* < 0.05) after the LPS challenged for 6 h and 3 h, respectively, compared with those in the LPS + PBS + MO/MΦ group. Meanwhile, the expression of *IL-1β* in the LTA + VIP + MO/MΦ group significantly decreased (*p* < 0.05) after LTA treated for 3 h, compared with the LTA + PBS + MO/MΦ group. Lastly, the expression levels of *IL-10* and *TGF-β* in the LTA + VIP + MO/MΦ group increased substantially (*p* < 0.05) after LTA treated for 6 h, compared with the LTA + PBS + MO/MΦ group (Figure 4).

### 2.5. Effects of On-VIP on Immune-Related Pathways in Tilapia MO/MΦ

**Figure 5:**
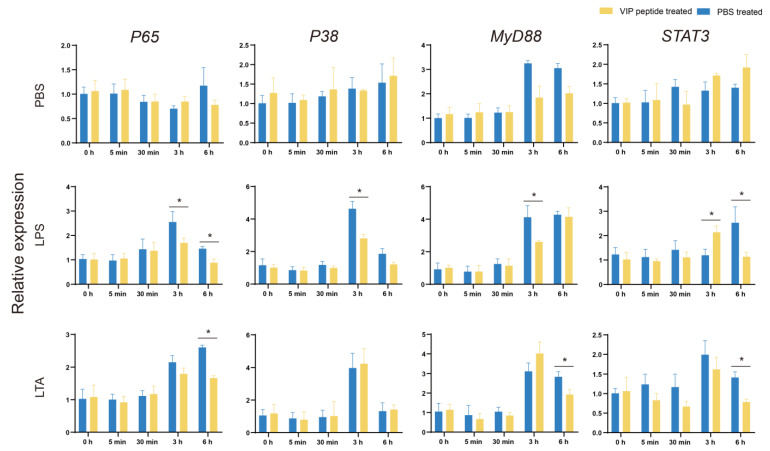
The relative expression of immune-related pathways (*P65*, *P38*, *MYD88*, and *STAT3*) after LPS and LTA challenges of tilapia MO/MΦ was assessed using qRT-PCR. Every value is presented as the mean and standard deviation; n = 3. The asterisks reveal the significant difference (*p* < 0.05).

The four typical factors (*P65*, *P38*, *MyD88,* and *STAT3*) were selected to investigate the probable mechanisms of VIP in tilapia MO/MΦ immunity regulation. The relative expression of *P65*, *P38*, and *MyD88* in the LPS + VIP + MO/MΦ group decreased significantly compared with those in the LPS + PBS + MO/MΦ group after 3 h of LPS challenges. Meanwhile, the similar results were observed for *P65*, *MyD88*, and *STAT3* in the LTA + VIP + MO/MΦ group after 6 h of LTA challenges (Figure 5).

### 2.6. Effects of On-VIP on cAMP-PKA Pathway in Tilapia MO/MΦ

**Figure 6:**
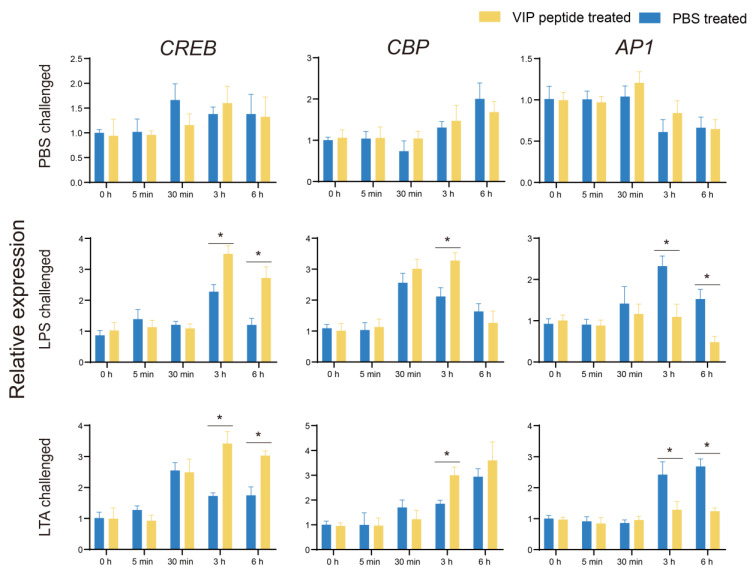
The expression profiles of cAMP-PKA pathway-related factors (*CREB*, *CBP*, and *AP1*) after LPS and LTA challenges of tilapia MO/MΦ were detected using qRT-PCR. Every value is presented as the mean and standard deviation; n = 3. The asterisks reveal the significant difference (*p* < 0.05).

The three key roles (*CREB*, *CBP*, *AP1*) of cAMP-PKA pathway were selected to investigate the probable mechanisms of VIP in tilapia MO/MΦ immunity regulation. The expression of *CREB* in the VIP treated group was mainly increased by On-VIP after LPS and LTA challenges 3 h and 6 h compared with those in the PBS treated group. Meanwhile, similar results were observed for *CBP* after LPS and LTA challenges 3 h compared the PBS treated group. However, *AP1* in the VIP-treated group significantly decreased after LPS and LTA challenges 3 h and 6 h compared to the PBS-treated group (Figure 6).

### 2.7. Effects of On-VIP on the Survival Rate and Bacterial Number of Tilapia under Bacterial Infection

**Figure 7:**
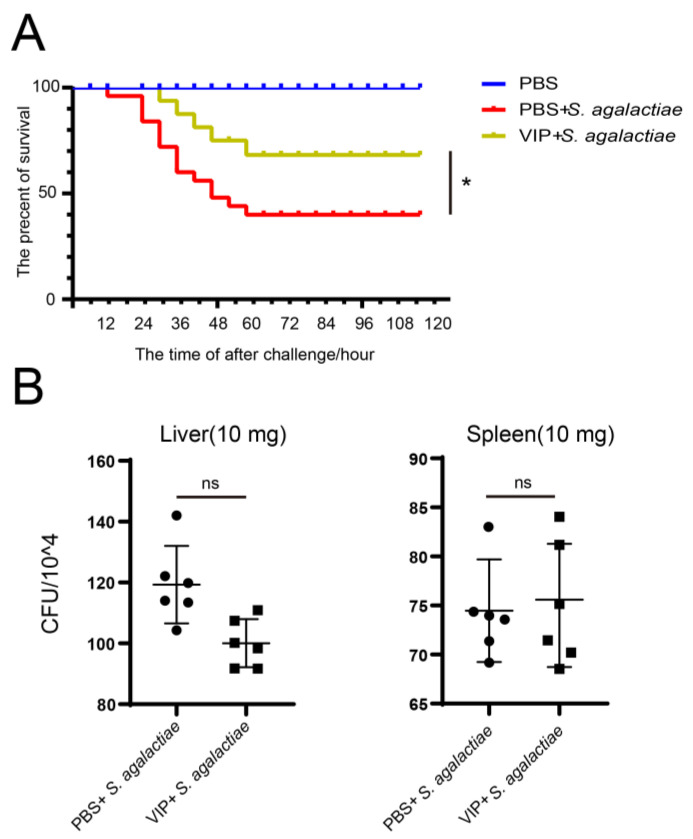
(**A**) After *S. agalactiae* infection, the survival rates of Nile tilapia. The PBS-treated Nile tilapia for control, deaths were counted every 6 h for a total of 120 h, n = 100 for each group. Each fish in the PBS group, PBS + *S. agalactiae* group, and On-VIP + *S. agalactiae* group was injected with 100 μL PBS, *S. agalactiae*, and mixture of *S. agalactiae* and On-VIP peptide, respectively. Asterisks indicate a significant difference (*p* < 0.05). (**B**) At 24 h after *S. agalactiae* infection, the bacterial loads of the liver and spleen were detected. Every value is presented as the mean and standard deviation; n = 6; ns is used to indicate no significant difference (*p* > 0.05).

The survival rates were 100%, 41%, and 68% for the PBS, PBS + *S. agalactiae*, and On-VIP + *S. agalactiae* groups, respectively. (Figure 7A). However, it was interesting that there was no significant difference in the number of bacteria belonging to PBS + *S. agalactiae* and On-VIP + *S. agalactiae* group in the liver and spleen. Meanwhile, the number of bacteria in the liver was more than that in the spleen (Figure 7B).

### 2.8. Effects of On-VIP on Inflammatory Factors of Tilapia under Bacterial Infection

**Figure 8:**
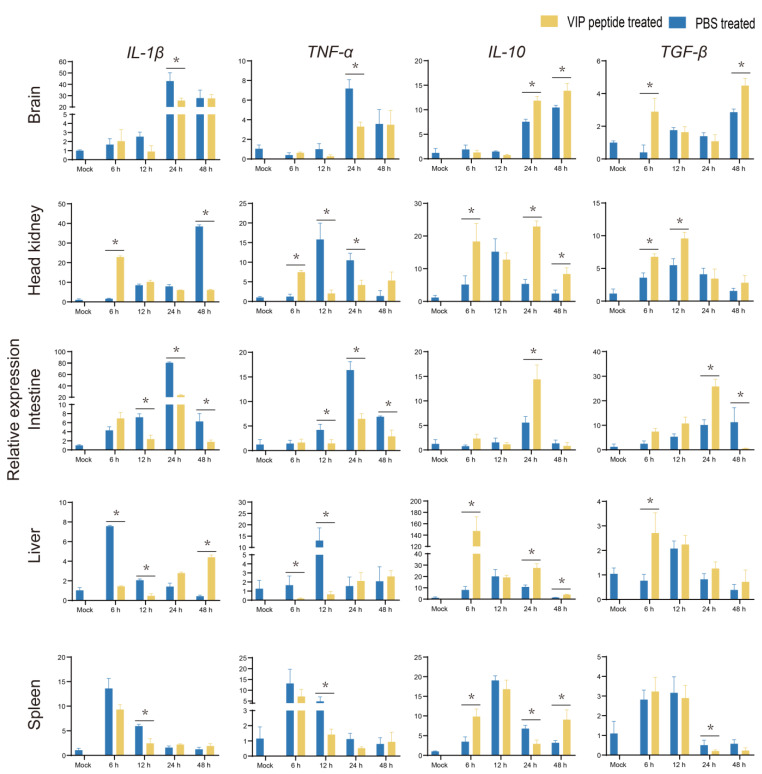
The expression patterns of inflammatory-related factors (*IL-1β*, *TNF-α*, *IL-10*, and *TGF-β*) at various points after *S. agalactiae* infection via qRT-PCR. Every value was presented as the mean and standard deviation; n = 3. The asterisks reveal the significant difference (*p* < 0.05).

In comparison to the PBS + *S. agalactiae* group, the transcriptional levels of pro-inflammatory factors (*IL-1β* and *TNF-α*) were significantly downregulated (*p* < 0.05) in most tissues from 6 to 24 h after the *S. agalactiae* challenged in the On-VIP + *S. agalactiae* group. However, at 6 h and 48 h post-challenge, the transcriptional levels of *IL-1β* in the On-VIP + *S. agalactiae* group increased significantly (*p* < 0.05) in comparison to PBS + *S. agalactiae* group in the liver. Additionally, contrasting results were observed in anti-inflammatory factors (*IL-10* and *TGF-β*), whose expression were mainly upregulated in the On-VIP + *S. agalactiae* group in comparison to the PBS + *S. agalactiae* group (Figure 8).

### 2.9. Effects of On-VIP on Immune-Related Pathways of Tilapia under Bacterial Infection

**Figure 9:**
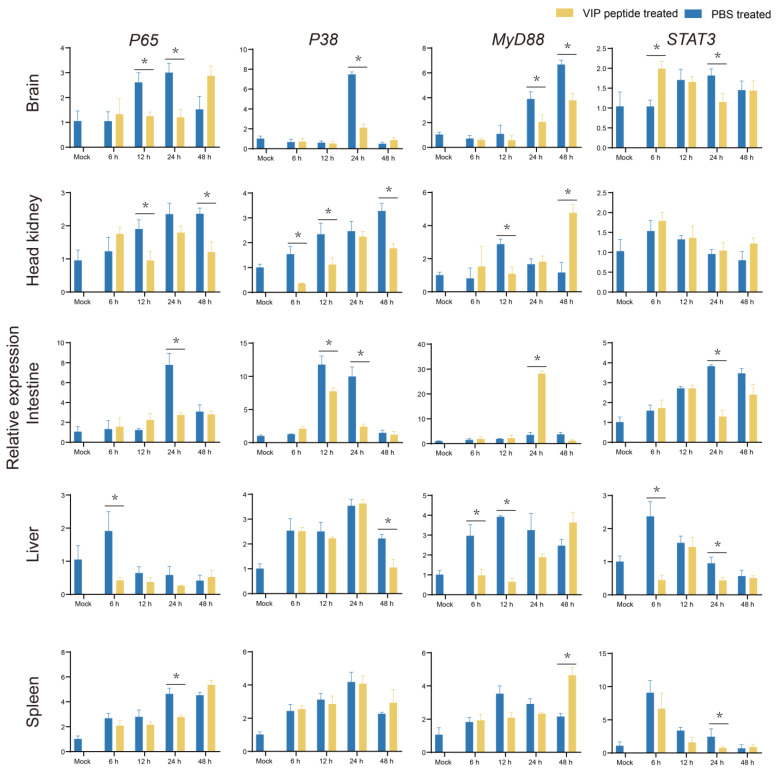
The expression profiles of immune-related pathways (*P65*, *P38*, *MYD88*, and *STAT3*) at various points after *S. agalactiae* infection via qRT-PCR. Every value is presented as the mean and standard deviation; n = 3. The asterisks reveal the significant difference (*p* < 0.05).

The transcriptional levels of *P65* in the On-VIP + *S. agalactiae* group showed a significant downregulation (*p* < 0.05) from 6 h to 48 h post-challenge in the brain, head kidney, intestine, liver, and spleen, compared to the PBS + *S. agalactiae* group during bacterial infection. Meanwhile, compared to the PBS + *S. agalactiae* group, similar results were observed for *P38* in the brain, head kidney, intestine, and liver of On-VIP + *S. agalactiae* group. Moreover, in comparison to the PBS + *S. agalactiae* group, the transcriptional levels of *MyD88* in the On-VIP + *S. agalactiae* group were markedly decreased (*p* < 0.05) in the brain and liver while the opposite result was observed in the intestine and spleen. Finally, the transcriptional levels of *STAT3* were downregulated by On-VIP in the intestine, liver, and spleen (Figure 9).

### 2.10. Effects of On-VIP on cAMP-PKA Pathway of Tilapia under Bacterial Infection

**Figure 10:**
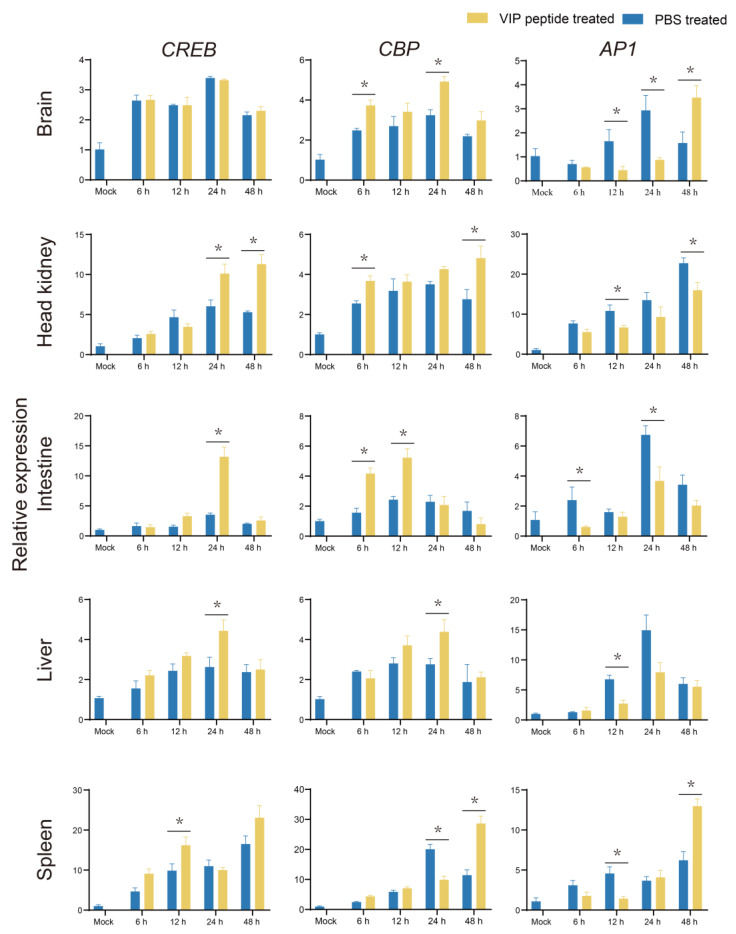
The expression profiles of cAMP-PKA pathway-related factors (*CREB*, *CBP*, and *AP1*) at various points after *S. agalactiae* infection via qRT-PCR. Every value is presented as the mean and standard deviation; n = 3. The asterisks reveal the significant difference (*p* < 0.05).

From 12 h to 48 h post-challenge, the transcriptional levels of *CREB* in the On-VIP + *S. agalactiae* group was markedly upregulated (*p* < 0.05) in the intestine, head kidney, spleen, and liver in comparison to the PBS + *S. agalactiae* group, while in the brain, *CREB* was not regulated by On-VIP. Meanwhile, the expression of *CBP* was markedly increased (*p* < 0.05) by On-VIP in the brain, head kidney, intestine, and liver. Moreover, in comparison to the PBS + *S. agalactiae* group, the transcriptional levels of *AP1* in the On-VIP + *S. agalactiae* group were markedly decreased (*p* < 0.05) in intestine, head kidney, and liver (Figure 10).

### 2.11. Effects of On-VIP on Apoptosis and Pyroptosis Factors of Tilapia under Bacterial Infection

**Figure 11:**
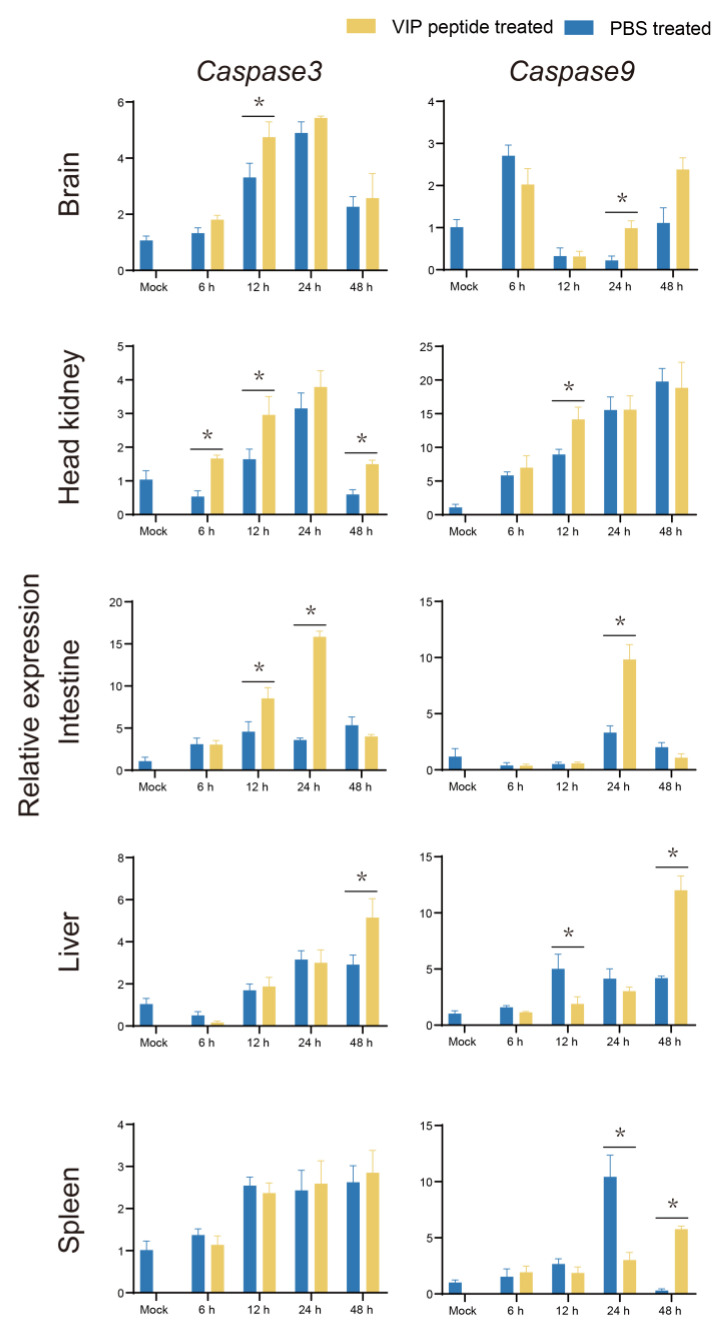
The expression profiles of classic apoptosis factors (*Caspase3* and *Caspase9*) at various points after *S. agalactiae* infection via qRT-PCR. Every value is presented as the mean and standard deviation; n = 3. The asterisks reveal the significant difference (*p* < 0.05).

**Figure 12:**
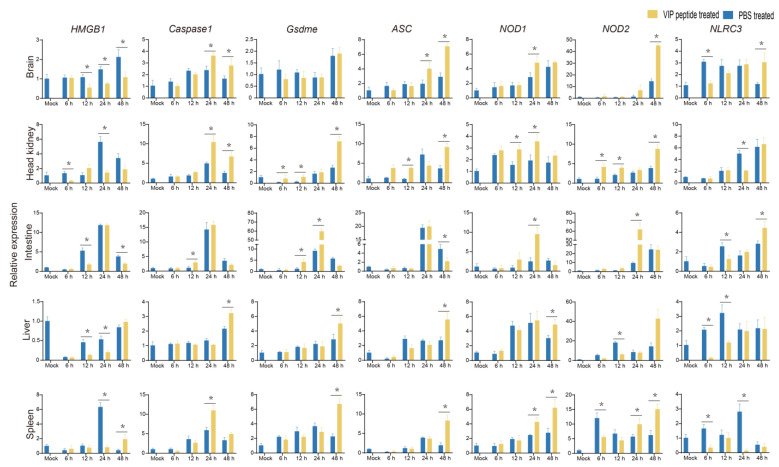
The relative expression of pyroptosis-related factors (*HMGB1, Caspase1, Gsdme, ASC, NOD1*, *NOD2*, and *NLRC3*) at various points after *S. agalactiae* infection via qRT-PCR. Every value is presented as the mean and standard deviation; n = 3. The asterisks reveal the significant difference (*p* < 0.05).

*Caspase3* and *Caspase9* (apoptosis-related factors) were investigated to evaluate the effect of On-VIP on apoptosis. Compared to PBS + *S. agalactiae* group, *Caspase3* and *Caspase9* in most selected organs of the On-VIP + *S. agalactiae* group were significantly increased (Figure 11). Seven key factors of pyroptosis were selected to investigate the influence of On-VIP on pyroptosis, namely, *HMGB1*, *Caspase1*, *Gsdme*, *ASC*, *NOD1*, *NOD2*, and *NLRC3*. In comparison to the PBS + *S. agalactiae* group, the expression of *HMGB1* was mainly downregulated by On-VIP. However, the opposite result was observed in *Caspase1*, *Gsdme*, *NOD1,* and*NOD2* in most tissues. Meanwhile, the expression of *ASC* was significantly upregulated (*p* < 0.05) by On-VIP in the brain, head kidney, liver, and spleen, but markedly downregulated (*p* < 0.05) in the liver. Moreover, *NLRC3* was downregulated in head kidney, liver, and spleen by On-VIP (Figure 12).

## 3. Discussion

As a vasodilator, VIP was initially isolated from the gastrointestinal tract in rats and humans [1,22]. Afterward, VIP was also found in the central nervous system (CNS) and participates in the regulation of immune activation as a neurotransmitter [3,23]. Moreover, a series of studies revealed that VIP was involved in various biological activities, such as hormonal regulation, hyperglycemia, inflammatory response, and systemic vasodilation [24,25]. The VIP and VIPR1 from Nile tilapia were identified and characterized in this study. The regulatory activities and mechanisms of VIP in the immune response of teleost against bacterial infection were initially investigated.

The On-VIP shares over 69% in common with other bony fish’s VIP, and contains a 25-amino acid signal peptide and a VIP peptides [26]. Although the multiple sequence alignment showed that On-VIP has a similar amino acid structure with mammals’ VIP, the amino acids between the signal peptide and VIP peptide were lost in fish. This finding suggests that VIP may have evolved over several generations in vertebrates. Moreover, high conservation was observed in the multiple sequence alignment of VIPR1. Specifically, the On-VIPR1 shares over 73% in common with other bony fish’s VIPR1 and more than 53% with mammals’ VIPR1, and On-VIPR1 has some similar structures (seven transmembrane domains) with mammals’ VIPR1. This finding implies the importance of VIP and VIPR1 during the evolution. Simultaneously, the importance of VIP-VIPR1 interaction amongst vertebrates and invertebrates has been proven because of their identification in *Leishmania braziliensis* [27]. Moreover, as a well-conserved glucagon/secretin from protochordate to mammals, On-VIP has shorter and leaner amino acid sequences compared with other mammals’ VIP, which may be due to the two rounds of genome duplications and diversification of vertebrate lineages [28]. The evolutionary tree of VIP and VIPR1 proved this suspicion and showed that On-VIP and On-VIPR1 were first grouped with bony fish and then with mammals.

The highest expression of On-VIP was investigated in the intestine, and the lower expression was detected in the brain and muscle, while the lowest was observed in the spleen. The similar transcriptional expression of VIP in dogfish shark and *Paralichthys olivaceus* has been proven [17,29]. However, the expression of On-VIP at the protein level was inconsistent with that at the transcription level, which may be related to the synthesis and release of neuropeptides, while the expression of On-VIPR1 at the protein level was consistent with that at the transcription level, and highest and lowest expressions were observed in the liver and skin, respectively. Nevertheless, the highest expression of VIPR1 was recorded in the brain of zebrafish [30]. This finding was not surprising since VIPR1 also shows high expression in the brain of Nile tilapia. The fluorescence immunohistochemistry was used to further investigate the interaction between VIP and VIPR1. In the muscular layer of longitudinal tissue sections of the duodenum of mice, specific immune signals of VIP were observed around the villus epithelium and into the lamina propria region [31]. The specific fluorescence immunohistochemistry signal for On-VIP in this study agrees with the results stated in the previous studies. IHC signal for VIPR1 was observed around the intestinal mucosa in humans [11]. Consistent with the previous study, specifically, the specific fluorescence IF signal for On-VIPR1 was revealed in the mucosal epithelium in our study. Similar expression patterns and localization imply that the function of VIP and VIPR1 may be relatively conserved in the intestine from bony Osteichthyes to mammals.

VIP, as a well-known endogenous anti-inflammatory neuropeptide, has shown potential in the treatment of various immune-related diseases [10,32]. Therefore, the model of bacterial infection in warm water was used to reveal the potential function of On-VIP in this study [33]. After the *S. agalactiae* challenge, the expression levels of *On-VIP* were upregulated in all tested tissues. Furthermore, *On-VIP* was quickly increasing with the extension of bacterial infection time in the head kidney and intestine, as well as a significant increase being observed at 6 h after infection. However, the expression levels of On-VIP were remarkably increased in the brain at 24 h after infection. Therefore, the VIP in the head kidney and intestine was upregulated faster than that in the brain, which was because the stress reaction induced by intraperitoneal injection was usually rapid in peripheral immune organs, such as the intestine, and sluggish in the brain [34,35]. Briefly, results implied that On-VIP was probably implicated in Nile tilapia’s immunological response to *S. agalactiae*, as well as showing that fish still have functional neuro-immune communication. Furthermore, the transcriptional expression of *On-VIPR1* was only upregulated at a few time points in the intestine, liver, and head kidney post challenge. Specifically, the transcriptional expression of On-VIPR1 maintains a constitutive expression in the brain and spleen. However, it was still implied that On-VIPR1 possibly reacts with On-VIP and as a complex involved in the immunization activities against *S. agalactiae* infection. The previous studies proved that VIP and VIPR1 play a crucial part in the control of immunity, inflammation, and pancreatic insulin secretion, and the release of catecholamines from the adrenal medulla in the periphery [36,37].

VIP could be produced by macrophages or T helper 2 cells in response to viral and pathogenic stimulation. Nerve terminals and blood may also release VIP [6]. The On-VIP peptide was gained and the MO/MΦ of tilapia were used as model for accessing the effects of On-VIP in vitro to further reveal the potential functions of VIP during immunization activities. A total of four genes of pro-/anti-inflammatory (*IL-1β*, *TNF-α*, *IL-10*, *TGF-β*), four key genes of immune-related pathways (*P65*, *P38*, *MyD88*, and *STAT3*), three key genes of cAMP-PKA pathway (*CREB*, *CBP*, *AP1*) were investigated via qRT-PCR. Consistent with a previous study, our results showed that the exogenous VIP may act as an anti-inflammatory agent inhibiting the expression of *IL-1β* and *TNF-α*, and enhanced expression of *IL-10* and *TGF-β* in LPS and LTA challenged tilapia MO/MΦ [10]. Moreover, our results showed that the NF-κB, MAPK, MyD88, and JNK-STAT pathways were inhibited by mature On-VIP peptide in LPS and LTA challenged tilapia MO/MΦ. The previous studies proved that VIP can inhibit the NF-κB transcriptional activity and STAT1 phosphorylation [38,39] and can decrease the expression of *P38* by inhibiting TATA-box-binding protein phosphorylation and reducing RNA pol II recruitment [40]. Additionally, VIP can downregulate the expression of *TLR4* and *MyD88*, but cannot induce constitutive expression [41]. In addition, VIP penetrates the cell membrane in combination with VIPR1 to further activate the camp-independent pathway, upregulate the expression of *CREB* and coactivator *CBP* in the cAMP-dependent pathway, and change the *AP1* composition and bind to the TNF-α promoter [38,42]. Likewise, the decreased expression of *AP1* mediated by VIP and the promoted expression of *CREB* and *CBP* were observed in our results, implying that vertebrates may have highly conservative regulatory mechanisms for VIP.

The mature On-VIP peptide was mixed with *S. agalactiae* and injected into Nile tilapia to further investigate the functions of VIP during the immunological response to bacteria in vivo. A considerable protective effect was observed in the VIP treatment group after *S. agalactiae* infection, but another interesting result was observed that the bacterial burden between PBS + *S. agalactiae* and VIP + *S. agalactiae* groups was not significantly different. The results implied that VIP may not enhance the eradication of harmful germs directly. Therefore, four inflammatory factors (*IL-1β*, *TNF-α*, *IL-10*, and *TGF-β*) and immune-related pathways (NF-κB, MAPK, MyD88, and JNK-STAT), three cAMP-PKA pathway related factors (*CREB*, *CBP*, and *AP1*), two classical apoptosis factors (*Caspase3* and *Caspase9*), and seven key factors of pyroptosis were assessed to study the roles of VIP on regulating immunity in vivo. In Nile tilapia, On-VIP may also act as an anti-inflammatory agent like that in mammals and inhibit the inflammatory response caused by bacterial infection [10]. The inhibited expression of *P65*, *P38,* and *STAT3* adjusted by On-VIP was observed in vivo infection, similar to that in vitro infection. While the regulation of On-VIP on the *MyD88* pathway was specific to tissues after bacterial infection, thus promoting its expression in the intestine and spleen and inhibiting its expression in the liver and brain. These results may be due to On-VIP existing in secretory pathways in these tissues such as those in mammals [43]. Meanwhile, VIP/PACAP inhibit NF-kappa B transactivation in the lipopolysaccharide-stimulated human monocytic cell line THP-1 at multiple levels have been reported [38]. Moreover, VIP inhibits JNK-STAT and MAPK pathway in the human also have been proved, and another study demonstrated that VIP performs a variety of physiological functions through cAMP-PKA pathways in vertebrate [10,38,39,40]. However, only one study about VIP in fish mentioned that several elements involved in cytokine-mediated activation are highly conserved in VIP of the 5′-flanking regions: cAMP responsive elements (CREs), binding sites for nuclear factor IL-6 (NF-IL-6), activating protein-1 (AP-1), stimulating protein-1 (Sp-1), two IL-6 responsive element binding proteins (IL-6 RE-BPs), and signal transducers and activators of transcription (STAT) [29]. Therefore, research on this bony fish is still lacking, and further research is necessary to identify the full range of possibilities for these results. Moreover, the results for the cAMP-PKA pathway were evaluated through in vivo infection and were consistent with those obtained through in vitro infection, suggesting that the immune regulation mechanisms of VIP may be conservative in Nile tilapia.

A sort of programmed cell death known as apoptosis, which happens in multicellular organisms, was a tightly controlled and regulated process that offers benefits throughout an organism’s life cycle [44,45]. A previous study showed that *Caspase-3*, one of the most typical factors of apoptosis, was significantly increased in Huh7 cells cultured with VIP [46]. Similarly, two typical factors of apoptosis (*Caspase-3* and *Caspase-9*) were significantly increased in this study at VIP treatment group. Pyroptosis was inherently inflammatory, was caused by various pathological stimuli, such as stroke, heart attack, or cancer, and plays a crucial role in controlling microbial infections [47]. The expression of a series of factors of pyroptosis, such as *Caspase1*, *Gsdme* (the executor), *ASC* (an adaptor), and *NOD1*/2 (the sensor proteins), were upregulated promptly 12 h post-challenge after VIP treatment whereas *HMGB1* was slightly downregulated after VIP treatment [48,49,50,51]. The different trend may be caused by the fact that *Caspase1* belongs to the canonical and noncanonical inflammasome pathways, respectively [52,53]. Meanwhile, *HMGB1*, a late-occurring cytokine, can be downregulated by VIP, as proven in mice, and was critical in endotoxemia and sepsis [54]. However, there have only been a few studies about pyroptosis in fish, and research is required to elucidate the relationship between VIP and pyroptosis in detail.

## 4. Materials and Methods

### 4.1. Fish Preparation

All Nile tilapia (50 ± 10 g) utilized in this work were acquired from a commercial fish farm in Zhanjiang city, Guangdong Province, China. The tilapia were cultured at a density of 50 fish per 1000 L water for 2 weeks and fed with the commercial mixed feed of about 1.5 g/fish daily. The temperature, pH, and dissolved oxygen of aquatic water were strictly maintained as mentioned in the previous study [33]. The fish used in subsequent experiments were randomly selected from a holding pond.

### 4.2. On-VIP Preparation

The mature On-VIP peptide (HTDAIFTDNYSRFRKQMAVKKYLNSVLS-NH2, molecular weight = 3332.8) was synthesized by Sangon Biotech (Shanghai, China), and the purity of the On-VIP peptide was 98%. Before use, On-VIP was dissolved with sterile phosphate-buffered saline (PBS) to obtain a concentration of 1 mg/mL (0.772 M).

### 4.3. S. Agalatiae Infection and Sample Collection

The *S. agalatiae* (ZQ0910) strains were kept in our lab and its complete genome sequence has been mentioned in previous work [55]. The strain was taken out from a −80 °C environment and cultured in liquid BHI medium (Huankai Microbial, Guangzhou, China) and incubator for 10 h at 28 °C. Subsequently, the *S. agalactiae* was collected by centrifugation and washed three times with PBS. A total of 50 healthy tilapias were collected and intraperitoneally injected with 100 μL/fish of *S. agalactiae* (5 × 10^7^ CFU/mL). The injected tilapias were initially narcotized with MS-222 (Sigma, Darmstadt, Germany) and the *S. agalactiae* sensitive tissues (brain, head kidney, intestine, liver, and spleen) were gained from three challenged fish at 0 h, 6 h, 12 h, 24 h, and 48 h. Moreover, the classical organs (brain, gill, head kidney, intestine, liver, muscle, skin, and spleen) from three unchallenged and healthy fish were collected as stated to investigate the tissue distribution of On-VIP and On-VIPR1.

### 4.4. Monocytes/Macrophages (MO/MΦ) Isolation and On-VIP Functions Assay In Vitro

The MO/MΦ of tilapia was isolated and challenged with LPS (Beyotime, Shanghai, China) and LTA (Sigma-Aldrich, St. Louis, MO, USA) to reveal the functions of On-VIP in vitro. Head kidney leukocytes (HKLs) were isolated from Nile tilapia following previous methods and cultured with L-15 medium (Gibco, New York, NY, USA) [35]. In brief, the head kidney was gained from three unchallenged and healthy fish was carefully cut into pieces and crossed a 40 μm stainless nylon mesh (Beyotime, Shanghai, China) to gain cells suspended in L15 medium. The cells were placed on top of 34%/51% percoll (Solarbio, Beijing, China) and centrifuged horizontally at 600 g for 30 min. The cells at the 51% percoll interface were harvested and gently washed three times with L15 medium. After that, 1 × 10^6^ cells/mL of HKLs were cultured in Gibco’s L-15 Medium for 24 h at 28 °C. The non-adherent cells were abandoned using the L-15 Medium suspended three times. The adherent MO/MΦ cells were separated by trypsin with 0.05% EDTA (Thermo Fisher Scientific, Waltham, MA, USA) and obtained by centrifugation (1000× *g*, 5 min). The collected MO/MΦ cells were washed three times, resuspended in PBS, and counted. The cells were divided into six groups with a final concentration of 1 × 10^6^ cells/well, were PBS + MO/MΦ, LPS + PBS + MO/MΦ, LTA + PBS + MO/MΦ, VIP + MO/MΦ, LPS + VIP + MO/MΦ, and LTA + VIP + MO/MΦ. For the PBS treated and VIP treated groups, 10 μl PBS and 0.1 μmol On-VIP peptide were added in MO/MΦ, respectively. The samples were collected at 0 h, 5 min, 30 min, 3 h, and 6 h after 10 μg LPS or LTA challenge.

### 4.5. RNA Extraction and cDNA Synthesis

The RNA extraction and cDNA synthesis of collected organs and MO/MΦ were performed following the manufacturer’s instructions of RNAiso Plus (TaKaRa, Dalian, China) and PrimeScript™ RT reagent kit with gDNA Eraser (TaKaRa, Dalian, China), respectively. Afterward, the redistilled water was used to dilute cDNA following our previous studies for subsequent experiments [34].

### 4.6. Cloning and Sequence Analysis of On-VIP and On-VIPR1

The predicted gene sequence of *On-VIP* and *On-VIPR1* was gained from the NCBI database, and the corresponding NCBI reference sequences were XM_005474399.4 and XM_003439191.5, respectively. A pair of particular primers and intestinal cDNA were used in PCR to amplify the *On-VIP* and *On-VIPR1* ORF sequences (Table S1).

The DNAMAN software was used as a vehicle for performing the multiple sequence alignments of VIP and VIPR1 between different species at protein levels. Meanwhile, the similarity of VIP and VIPR1 between different species was indicated via NCBI Blast (https://blast.ncbi.nlm.nih.gov/BlastAlign.cgi, accessed on 13 May 2022). The MEGA software (version 6.0) was used to construct the phylogenetic tree via neighbor-joining (NJ) methods. The signalP was used to predict the potential signal peptide (http://www.cbs.dtu.dk/services/SignalP/, accessed on 13 May 2022). The TMHMM was used to predict the transmembrane domain of On-VIP and On-VIPR1 (https://services.healthtech.dtu.dk/service.php?TMHMM-2.0, accessed on 13 May 2022). The On-VIP and On-VIPR1’s molecular weight, amino acid composition, and theoretical p*I* were forecasted using the ProtParam tool (https://web.expasy.org/protparam/, accessed on 13 May 2022).

### 4.7. Quantitative Real-Time PCR (qRT-PCR)

The tissue distributions of *On-VIP* and *On-VIPR1* at the mRNA level in unchallenged and healthy tilapia and the expression patterns after *S.agalactiae* infection were assessed using the QuantStudio 6 Flex Real-Time PCR Systems (Thermo Fisher Scientific, Waltham, MA, USA) and TB Green^®^ Premix Ex Taq™ II (Tli RNaseH Plus) (TaKaRa, Dalian, China). The detailed reaction system was established as mentioned in our previous studies [34]. The transcriptional levels of *On-VIP* and *On-VIPR1* were computed using the previous method whereby *β-actin*, *gapdh*, and *ef1a* were selected as reference genes [56,57].

### 4.8. Western Blot

The tissue distributions of On-VIP and On-VIPR1 at the protein levels in health tilapia were investigated using Western blot. As previously mentioned, the brain, gills, head kidney, spleen, intestine, muscle, skin, and liver were removed from undisturbed and healthy tilapia, and the total protein was extracted using a protein extraction kit (Solarbio, Beijing, China). All total protein samples were processed by 6× Protein Loading Buffer (TRANS, Beijing, China) before performing Western blot.

For each organ, 25 μg total protein was added in 10% SDS-PAGE for electrophoresis at 200 V for 25 min, and transformed into a 0.45 μm PVDF membrane (Millipore) by dry rotary apparatus (Bio-rad, Hercules, CA, USA). The QuickBlock™ Blocking Buffer (Beyotime, Shanghai, China) was used to block PVDF membranes for 30 min at 28 °C. PVDF membranes were incubated for 40 min at 28 °C with the primary antibody anti-VIP antibody (bs-0077R, Bioss, Beijing, China) and anti-VIPR1 antibody (BA1462-2, BOSTER, Wuhan, China), respectively. Meanwhile, the anti-β-Actin antibody (AC026, ABclonal, Wuhan, China) was performed as a control. Afterward, every PVDF membrane was washed carefully using a mixture of TBS+0.1% Tween-20 (TBST) and incubated for 30 min at 28 °C with the secondary antibody goat anti-rabbit IgG (H+L) that has been labeled by HRP (Beyotime, Shanghai, China). Lastly, the PVDF membrane was thoroughly rinsed in TBST again, and then the antigen-antibody complexes were observed using Ultra-sensitive ECL Chemiluminescence Kit (P0018S, Beyotime, Shanghai, China). In addition, the dilution ratio of anti-VIP and anti-VIPR1 antibodies was 1:1000 and the dilution ratio of goat anti-rabbit IgG (H+L) labeled by HRP was 1:2000.

### 4.9. Fluorescent Immunohistochemistry

The histological observation was performed as in our earlier research [34]. In brief, the intestine was obtained from healthy tilapia as mentioned above. The collected intestines were fixed in Dietrich’s stationary liquid for 48 h and dehydrated by the gradient of ethanol (50%, 70%, 85%, 95%, and 100%) [34]. Afterward, samples were embedded in paraffin wax after vitrification by xylene. Intestinal sections with a thickness of 5 μm were dewaxed with xylene and then rehydrated by the gradient of ethanol (100%, 95%, 80%, 70%, and 50%). Samples were then treated with EDTA epitope repair solution (Beyotime, Shanghai, China) for 40 min at 98°C successively. Subsequently, samples were blocked using the QuickBlock™ Blocking Buffer for 30 min at 28 °C and rinsed twice. Afterward, samples were placed into the diluent of the anti-VIP antibody and anti-VIPR1 antibody and incubated for 1 h at 28 °C. Then, the former samples were rinsed five times and placed into a diluent of Cy3 goat anti-rabbit IgG (H+L) (AS007, ABclonal, Wuhan, China) for 1.5 h at 28 °C without light.

The former samples were then placed in 1 μg/mL DAPI (Beyotime, Shanghai, China) working solution for 5 min after washing five times with PBS. Finally, sections were washed with PBS, and imaging was done using the ZEISS Axioscope 5 microscope (Zeiss, Jena, Germany).

### 4.10. On-VIP Function and Molecular Mechanism Assay In Vivo

A total of 300 healthy tilapia were divided equally into three groups (i.e., PBS, PBS + *S. agalactiae,* and On-VIP + *S. agalactiae*). For the PBS group and PBS + *S. agalactiae*, 100 μL sterile PBS and *S. agalactiae* (5 × 10^7^ CFU/mL) was intraperitoneally injected into per fish, respectively. For the On-VIP + *S. agalactiae* group, 100 μL mixture of *S. agalactiae* and 16.66 μg (5 nmol) On-VIP peptide was injected into each fish.

For each group, the immune-related organs (brain, head kidney, intestine, liver, and spleen) were selected to extract total RNA and synthesize cDNA. About 10 mg organs (liver and spleen) of PBS + *S. agalactiae* and On-VIP + *S. agalactiae* groups were collected, broken, and dissolved in 1 mL sterile PBS at 48 h. Subsequently, samples were mixed with sterile PBS at a ratio of 1:1000, and 100 μL solutions were spread on BHI and cultured at 28 °C for 24 h. Finally, the bacterial species of 10 single colonies were verified using 16s rRNA genes and the CFU of *S. agalactiae* was counted.

Following our prior investigations, the tilapia mortality was determined six days after the challenge, and SR was computed [35].

The genes of pro-/anti-inflammatory (*IL-1β*, *TNF-α*, *IL-10*, *TGF-β*), four key genes of immune-related pathways (*P65*, *P38*, *MyD88*, and *STAT3*), three key genes of cAMP-PKA pathway (*CREB*, *CBP*, A*P1*), two apoptosis-related genes (*Caspase3*, *Caspase9*), and seven pyroptosis-related factors (*HMGB1*, *Caspase1*, *Gsdme*, *ASC*, *NOD1*, *NOD2*, *NLRC3*) were selected to reveal the roles of On-VIP and On-VIPR1 in the immune response against *S. agalactiae* (Table S1).

### 4.11. Animal Ethics

All experiments were carried out following the principles and procedures of the Regulations of Guangdong Province on the Management of Experimental Animals and approved by the Guangdong Ocean University’s Ethics Committee.

### 4.12. Drawings and Statistical Analysis

All data were shown as the mean with standard deviation, and the significant difference was analyzed using the one-way ANOVA (Tukey HSD test) and Student’s *t*-test through the Prism software (Version 8.0, Prism Software Company, Nashville, TN, USA). Meanwhile, the statistically significant differences (*p* < 0.05) were revealed by different letters or asterisks. Lastly, using Adobe Photoshop CC (San Jose, CA, USA) and Adobe Illustrator (San Jose, CA, USA), the drawings and final panel design were performed.

## 5. Conclusions

In summary, the VIP and its receptor VIPR1 homolog of Nile tilapia were identified, and the irreplaceable roles of On-VIP in the immunization activities of tilapia against *S. agalactiae* infection were determined in this study. On-VIP acts as a neurotransmitter and was widely distributed in many tissues of unchallenged and healthy tilapia. Meanwhile, remarkable On-VIP positive signals were observed in the longitudinal muscle layer of the healthy tilapia intestine, while On-VIPR1 was predominantly detected in the mucosal epithelium. In vitro experiments indicated several immune functions of On-VIP in Monocytes/Macrophages, including reduction of *P65*, *P38*, *MyD88*, *STAT3*, and *AP1*, upregulation of *CREB* and *CBP*, and suppression of inflammation. Moreover, in vivo experiments demonstrated that On-VIP could downregulate the expression of *P65*, *P38*, *MyD88*, *STAT3*, and *AP1*, reduce inflammation, upregulate the expression of *CREB* and *CBP*, improve survival rate, and promote apoptosis and pyroptosis. Therefore, On-VIP may play a crucial role in promoting healthy aquaculture. This study not only establishes a theoretical basis for further studies on the effects of neuropeptide in fish immunization activities on bacterial infection but also is significant for studies in ichthyopathology and the development of healthy aquaculture due to the importance of the species of Nile tilapia.

## Data Availability

The datasets presented in this study can be found in online repositories. The names of the repository/repositories and accession number(s) can be found in the article/supplementary material.

## Supplementary Materials

The following supporting information can be downloaded at: https://www.mdpi.com/article/10.3390/ijms232314895/s1.
